# Exercise-Induced Fluid Retention, Cardiac Volume Overload, and Peripheral Edema in Ultra-Distance Cyclists

**DOI:** 10.1016/j.ekir.2023.10.025

**Published:** 2023-10-31

**Authors:** Philipp Gauckler, Jana S. Kesenheimer, Johannes Leierer, Maren Kruus, Michael Schreinlechner, Fabian Theurl, Axel Bauer, Sara Denicolò, Alexander Egger, Beata Seeber, Gert Mayer, Fiona R. Kolbinger, Andreas Kronbichler

**Affiliations:** 1 Department of Internal Medicine IV (Nephrology and Hypertension), Medical University Innsbruck, Innsbruck, Austria; 2 Department of Psychology, University of Innsbruck, Innsbruck, Austria; 3 Department of Internal Medicine III (Cardiology and Angiology), Medical University Innsbruck, Innsbruck, Austria; 4 Central Institute of Medical and Chemical Laboratory Diagnostics (ZIMCL), University Hospital of Innsbruck, Innsbruck, Austria; 5 Department of Gynecologic Endocrinology and Reproductive Medicine, Medical University of Innsbruck, Innsbruck, Austria; 6 Department of Visceral, Thoracic and Vascular Surgery, University Hospital and Faculty of Medicine Carl Gustav Carus, TUD Dresden University of Technology, Dresden, Germany

**Keywords:** arginine vasopressin, exercise, fluid overload, overhydration, peripheral edema, ultra-endurance cycling

## Abstract

**Introduction:**

Ultracyclists expose themselves to extreme physical challenges. This study aimed to elucidate the effects of ultracycling on electrolyte and fluid balance and investigate the potential occurrence of peripheral edema.

**Methods:**

A total of 4 clinical visits were performed before, during, and after a 6-day bicycle ride in 13 ultracyclists (5 female, 8 male) including serial laboratory analyses of blood and urine, bioelectrical impedance, and echocardiography. Throughout the ride, participants continuously tracked fluid intake, measured extremity circumferences daily, and self-tested urinary electrolytes using a point-of-care testing device. Portrait photos were judged by 20 physicians for occurrence of facial and eyelid edema.

**Results:**

Participants covered a mean distance of 1205 km and 19,417 vertical meters. From baseline to day 6, body weight remained stable (*P* = 0.479); however, body composition changed with increasing total body water (TBW) (+1.98 l ± 1.37, *P* = 0.003) and plasma volume (+18.86 % ± 10.7, *P* < 0.001). A significant increase in N-terminal pro brain natriuretic peptide (NT-proBNP) (+297.99 ng/l ± 190.42, *P* < 0.001) until day 6 indicates concomitant cardiac volume overload. Swelling of face and eyelids peaked on day 5 (both *P* ≤ 0.033). On recovery, changes partly resolved. Although urinary sodium concentration showed a nadir on day 4 (−32.18 mmol/l ± 23.88, *P* = 0.022), plasma osmolality (+5.69 mmosmol/kg ± 5.88, *P* = 0.004) and copeptin (+38.28 pg/ml ± 18.90, *P* < 0.001) increased steadily until day 6.

**Conclusion:**

Ultracycling over multiple days induces extracellular volume expansion, peripheral edema, and cardiac volume overload. Renal sodium and water retention is likely contributing to this condition.

## Graphical abstract

**Figure:**
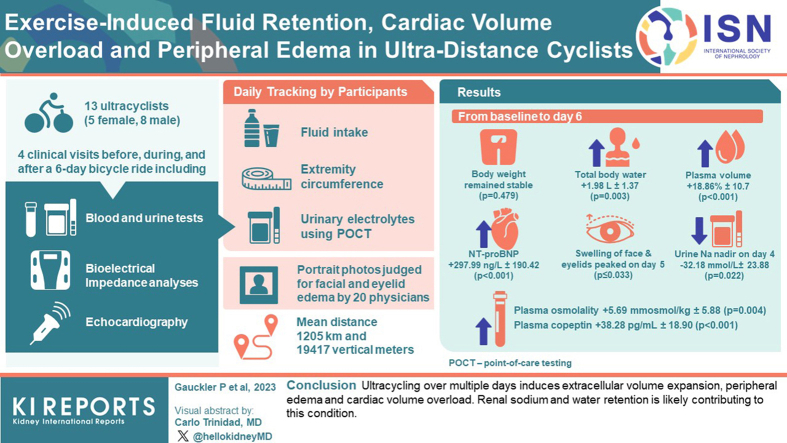

The popularity of ultraendurance cycling (ultracycling) is steadily increasing, reflected by increasing participant numbers in ultracycling events over the last decades.^1^ Although a uniform definition is lacking, ultracycling races usually cover distances of several hundred kilometers.^2^ Most ultracycling races last several days and athletes confront extreme physical and mental conditions, including sleep deprivation,^3^ and many ultracycling races do not provide any outside support.^4^ Such extreme conditions demand physiological adaptation processes involving different organ systems with potentially health-threatening consequences even in otherwise healthy athletes.^5^

We recently reported a high incidence of “swelling symptoms” in a representative cohort of ultracyclists. Among survey participants, 54.2% reported having experienced swelling symptoms of any body part during or after an ultracycling activity.^6^ Single cases of peripheral edema have been reported in ultratriathletes and ultramarathoners, mainly in the context of excess fluid intake.^7^^,^^8^ However, the underlying pathophysiology has not yet been studied systematically,^9^ and to the best of our knowledge, no cases have been reported in ultracyclists.

Although historically, much attention was paid to avoid dehydration, overhydration may be equally relevant.^10^ So far, the latter has mostly been discussed in the context of exercise-associated hyponatremia and excess fluid intake,^11^^,^^12^ which however is regarded rather rare in cycling sports.^13^ Drinking over thirst and nonosmotic release of arginine vasopressin (AVP) are 2 major drivers claimed to be responsible for the fluid volume excess observed in exercise-associated hyponatremia.^14^^,^^15^ Although the renin-angiotensin-aldosterone system (RAAS) is the main regulator of circulating volume to ensure adequate tissue perfusion, AVP release is primarily regulated by osmoreceptors effecting free water reabsorption in the renal collecting ducts. As the major effective extracellular solute, serum sodium concentration is the primary osmotic determinant of AVP release, whereas ineffective osmoles such as urea do not affect AVP secretion. However, several nonosmotic triggers of AVP release have been described, which may even overrule osmotic regulation in certain conditions.^16^

In this prospective, observational pilot study, we aimed to investigate the occurrence of peripheral edema and study the dynamics and underlying mechanisms of fluid and electrolyte balance in 13 experienced ultracyclists.

## Methods

### Participants

Five female and 8 male ultracyclists from 7 European countries were recruited via social media platforms. Inclusion criteria were the following: (i) age between 18 and 60 years and (ii) successful completion of at least 1 official ultraendurance cycling event. Participants were excluded if baseline diagnostics implied a health condition (e.g., cardiac alterations, chronic kidney disease, and pregnancy) that could make the planned physical activity harmful. To ensure privacy and data protection, demographic information of study participants was limited to the variables presented in Supplementary Table S1. Before the study, all 13 participants provided written informed consent.

### Study Course and Procedures

Study participants were invited to conduct an unsupported training bike ride over multiple days. Participation was for study purposes only. Each participant was free to choose a route, aiming to cover a distance of at least 1000 km and 10,000 m of elevation.

A comprehensive diagnostic approach was applied to assess the occurrence of peripheral edema and analyze potentially underlying mechanisms. The study timeline is presented in Supplementary Figure S1. A detailed description of all procedures, including physical and laboratory examinations as well as self-assessments and supplementary references are provided in the Supplementary Information. Clinical visits were performed at baseline (day 0), interim (day 4), immediately after the ride (day 6) and after 12 to 24 hours of recovery (day 7). Serum copeptin was analyzed as a surrogate marker for AVP using an enzyme-linked immunosorbent assay. Occurrence of peripheral edema of the extremities was assessed by measuring changes of body part circumferences and clinical judgement. Facial and eyelid edema were assessed through longitudinal photo documentation of each participant and judgement by 20 physicians.

This study was performed in line with the principles of the Helsinki Declaration of 1975, as revised in 2013. Approval was granted by the Ethics Committee of the Medical University of Innsbruck (1068/2021).

### Analysis

In the first step, we summarized relevant parameters using descriptive metrics. Relative changes over time were calculated and statistical significance of changes was tested using Wilcoxon tests (all tests of differences refer to Wilcoxon *P*-values, as a nonparametric option for the test of differences for dependent samples). To display parameter changes over time, we plotted smoothed regression lines using the R package ‘ggplot2.’^17^ Daily tracked body part circumferences and liquid intake were analyzed using multilevel regression models to address the nested data structure containing 2 levels: the individual changes (level 1) across 8 points of time (level 2), using the R packages ‘nlme’^18^ and ‘multilevel.’^19^

## Results

Thirteen ultracyclists (5 female, 8 male; mean age: 33.39 years, SD = 8.52) participated in the pilot study between September 4 and September 9, 2021. Baseline characteristics of riders and their riding performance are presented in Supplementary Table S1.

### Exercise-Induced Increase in Body Water

#### Changes of Body Composition

**Figure 1:**
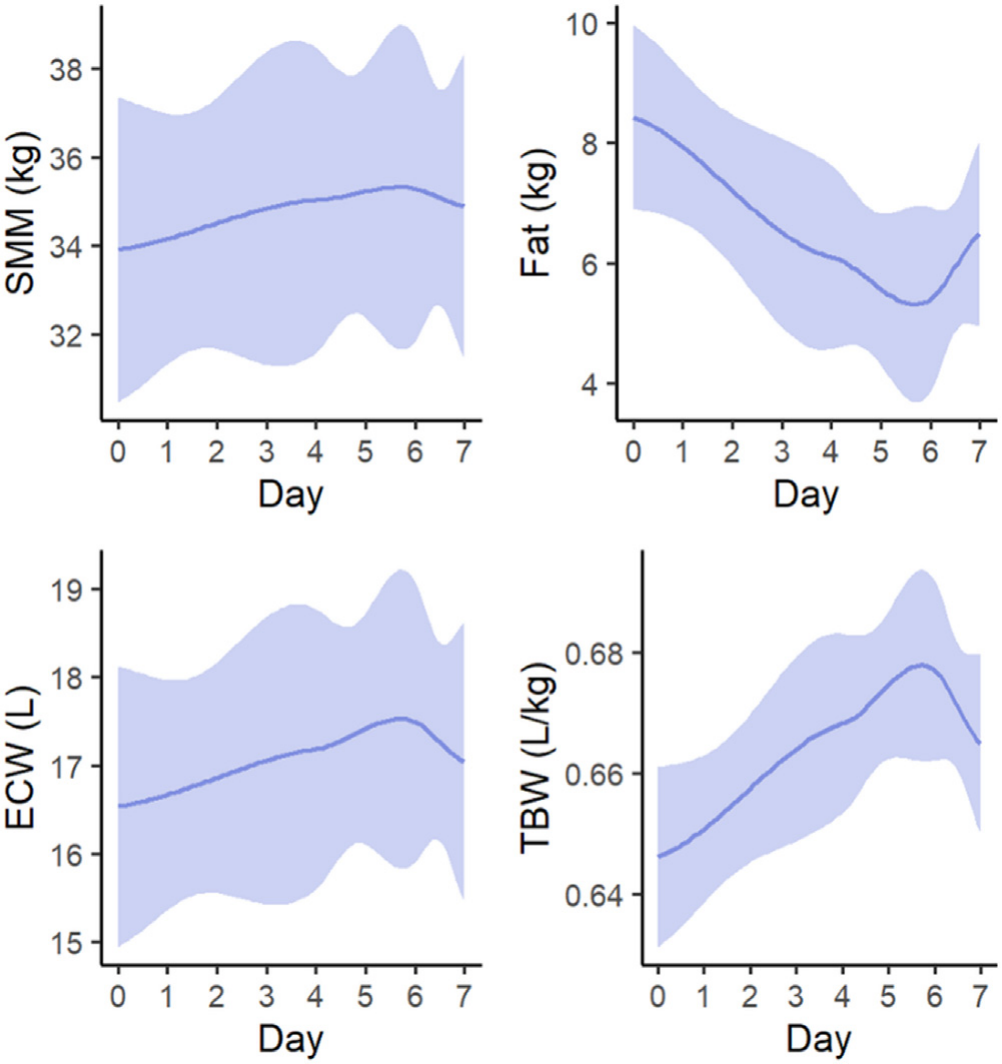
Development of BIA parameters over the course of the ride. The shaded area displays the 95% confidence interval. BIA, bioimpedance analysis; ECW, extracellular water; SMM, skeletal muscle mass; TBW, total body water.

Changes in body composition of 12 participants over the study course were analyzed using bioelectrical impedance analysis (BIA) (Supplementary Table S2). Because of a technical error that occurred in 2 measurements of 1 participant (ID 11), BIA results of this participant were excluded from further analysis. From day 0 to day 6, the mean body weight of the participants remained approximately stable (−0.183 kg ± 1.17, *P* = 0.479; −0.26%). The mean skeletal muscle mass increased by +1.38 kg ± 1.09 (*P* = 0.003), whereas the mean total fat mass decreased by −3.04 kg ± 2.71 (*P* = 0.003). Both TBW and extracellular water (ECW) significantly increased by +1.98 l ± 1.37 (*P* = 0.003) and +0.96 l ± 0.60 (*P* = 0.003), as displayed in Figure 1.

#### Increase in Plasma Volume and Cardiac Volume Overload

**Figure 2:**
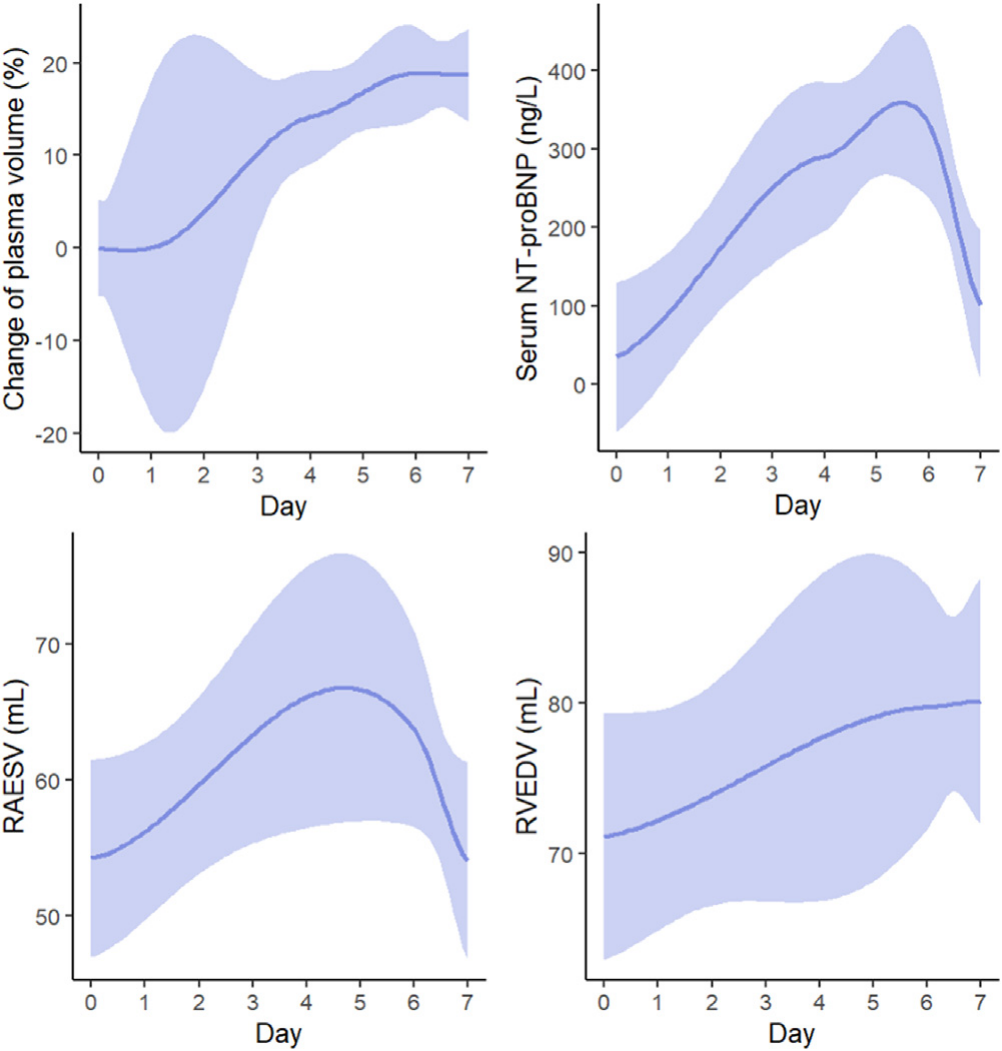
Change of plasma volume and NT-proBNP (upper panels) and cardiac volume changes in serial TTE examinations (lower panels). The shaded area displays a 95% confidence interval. NT-proBNP, N-terminal pro brain natriuretic peptide; RAESV, right atrial end-systolic volume; RVEDV, right ventricular end-diastolic volume; TTE, transthoracic echocardiography.

Changes in plasma volume and NT-proBNP during the study are illustrated in Figure 2 and summarized in Supplementary Table S3. Compared to baseline, plasma volume constantly increased during the ride, peaking on day 6 with a mean increase of +18.86% ± 10.7 (*P* < 0.001), and only a minimal recovery effect (+18.67% ± 8.69 compared to baseline, *P* < 0.001) on day 7. NT-proBNP peaked on day 6 (*M* = 332.46 ng/l ± 217.06) with a mean increase by 298 ng/l compared to baseline (*M* = 34.46 ng/l ± 18.87; *P* < 0.001) and returned to 100.54 ng/l ± 76.33 after recovery (on day 7), (*P* = 0.004) in comparison to day 6. There was no sign of myocardial damage as indicated by high-sensitivity troponin T values, which were within the normal range throughout the study (Table 1).

**Table 1:** Temporal changes in kidney-specific laboratory parameters over the course of the study bike ride

Variable	min/max	day			
		0 (pre)	4 (interim)	6 (post)	7 (recovery)
Creatinine (mg/dl)	0.59/1.13	0.80 (0.12)	0.82 (0.11)	0.86 (0.15)	0.77 (0.12)
eGFR (CKD-EPI Creatinine) (ml/min per 1.73 m^2^)	89.00/129.00	114.69 (11.64)	113.0 (9.17)	108.62 (12.24)	116.54 (10.78)
Cystatin C (mg/l)	0.65/1.01	0.82 (0.10)	0.80 (0.09)	0.82 (0.09)	0.82 (0.10)
eGFR (CKD-EPI Cystatin C) (ml/min per 1.73 m^2^)	87.00/128.00	107.00 (12.41)	110.31 (12.92)	108.62 (13.37)	107.46 (13.25)
Creatinine Clearance (ml/min)^a^	82.90/217.20	156.26 (29.12)	*NA*	*NA*	154.54 (21.57)
NGAL (μg/l)	25.00/169.00	50.62 (27.85)	57.39 (28.92)	62.39 (44.01)	55.69 (40.02)
Sodium (mmol/l)	133.00/142.00	136.77 (2.68)	137.92 (1.66)	138.54 (1.39)	137.31 (1.18)
Potassium (mmol/l)	3.30/5.10	4.31 (0.29)	3.71 (0.36)	4.04 (0.27)	3.99 (0.17)
Total Osmolality (mosmol/kg)	281.00/338.00	289.85 (5.54)	292.15 (4.16)	295.54 (3.31)	293.39 (13.99)
**Δ** Total Osmolality (mosmol/kg)		ref	+2.3	+5.69	+3.54
**Δ** Effective Osmolality (**Δ**sodium ∗2)		ref	+2.3	+3.54	+1.08
Copeptin (pg/ml)	11.39/147.14	34.03 (18.46)	55.10 (22.92)	72.31 (28.76)	60.87 (22.32)
Colloid osmotic pressure (mmHg)	21.00/31.70	27.03 (2.01)	24.55 (1.87)	24.35 (1.32)	22.66 (1.32)
Total protein (g/dl)	6.31/7.80	7.02 (0.29)	7.00 (0.34)	6.74 (0.22)	6.72 (0.26)
Albumin (mg/dl)	3650.00/4627.00	4282.85 (173.50)	4097.38 (245.65)	4207.31 (272.25)	4080.00 (294.06)
Creatine Kinase (U/l)	70.00/1516.00	201.46 (199.34)	447.54 (352.05)	382.54 (359.82)	296.92 (292.26)
hs Troponin T (ng/l)	2.50/12.40	4.10 (1.86)	6.90 (3.15)	6.09 (2.82)	5.02 (2.77)
C-reactive protein (mg/dl)	0.03/1.98	0.21 (0.26)	0.59 (0.54)	0.32 (0.35)	0.27 (0.28)
URINE					
Sodium (mmol/l) - POCT	0.40 /208.50	89.81 (44.99)	57.63 (43.35)	94.66 (43.63)	113.13 (46.59)
Potassium (mmol/l)	13.00/123.00	48.85 (24.03)	52.85 (17.48)	60.85 (26.70)	48.62 (24.83)
Osmolality (mosmol/kg)	192.00/1246.00	656.85 (295.04)	906.92 (283.37)	817.54 (162.60)	688.69 (207.55)
Na^+^/K^+^ ratio		1.84	1.10	1.56	2.33

CK, creatinine kinase; CKD-EPI, Chronic Kidney Disease Epidemiology Collaboration; COP, colloid osmotic pressure; eGFR, estimated glomerular filtration rate; hs, high-sensitivity; NGAL, neutrophil gelatinase-associated lipocalin; POCT, point-of-care testing.

Table displays mean values and standard deviations (in parentheses).

a 24-hour urine collection was only performed on days 1–2 and 6–7.

Transthoracic echocardiography examinations on days 0, 6, and 7 revealed signs of cardiac volume overload. End-systolic volume of the right atrium increased from 54.21 ml ± 11.25 at baseline to 63.84 ml ± 13.18 on day 6 (*P* = 0.058) and recovered completely on day 7 (54.00 ml ± 14.31, *P* = 0.878). End-diastolic volume of the right ventricle showed a steady increase from 71.09 ml ± 13.98 at baseline to a peak volume of 80.11 ml ± 14.42 on day 7 (*P* = 0.204) (Figure 2, Supplementary Table S4).

### Investigation of Peripheral Edema

#### Face and Eyelids

**Figure 3:**
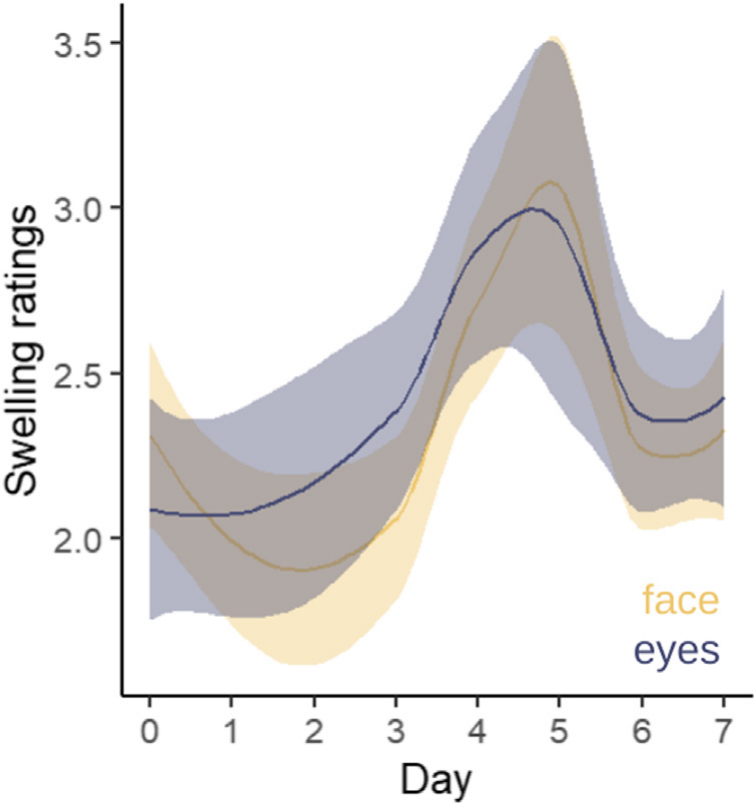
Swelling ratings of eyelids (blue line) and the whole face (yellow line) over time. The shaded area displays the 95% confidence interval.

Based on the assessment of participant portraits, blinded for the time point of collected, by 20 physicians, face and eyelid swelling increased from day 0 (face mean = 2.29 ± 0.35; eyelids mean = 2.07 ± 0.51) and peaked on day 5 (face mean = 3.06 ± 0.60, *P* = 0.033; eyelids mean = 2.94 ± 0.79, *P* = 0.030). The swelling recovered almost completely on day 7 (face mean = 2.38 ± 0.58, *P* = 0.857; eyelids mean = 2.46 ± 0.46, *P* = 0.021, Supplementary Table S5). An overall trend of the swelling ratings is illustrated in Figure 3.

#### Peripheral Edema

Obvious peripheral pitting edema was not detected by clinical judgment. Serial measurements of extremity circumferences are summarized in Supplementary Table S6. Multilevel models of serial body measurements showed a significant circumference increase over time (8 days) for the ankle (*beta* = 0.09, 95% confidence interval [CI]: 0.05–0.13, *t*(84) = 4.17, *P* < 0.001), wrist (*beta* = 0.04, 95% CI: 0.02–0.07, *t*(89) = 3.39, *P* = 0.001), thigh (*beta* = 0.15, 95% CI: 0.07–0.22, *t*(88) = 3.79, *P* < 0.001), and phalanx Dig. 2 (*beta* = 0.02, 95% CI: 0.01–0.03, *t*(89) = 3.16, *P* = 0.002). Other body part circumferences did not change significantly over time (calf: *beta* = 0, 95% CI: −0.07 to 0.06, *t*(88) = −0.12, *P* = 0.908; mid forearm: *beta* = 0.06, 95% CI: −0.01 to 0.13, *t*(89) = 1.58, *P* = 0.117; and mid upper arm: *beta* = −0.01, 95% CI: −0.07 to 0.05], *t*(89) = −0.32, *P* = 0.747).

### Pathomechanistic Considerations

**Figure 4:**
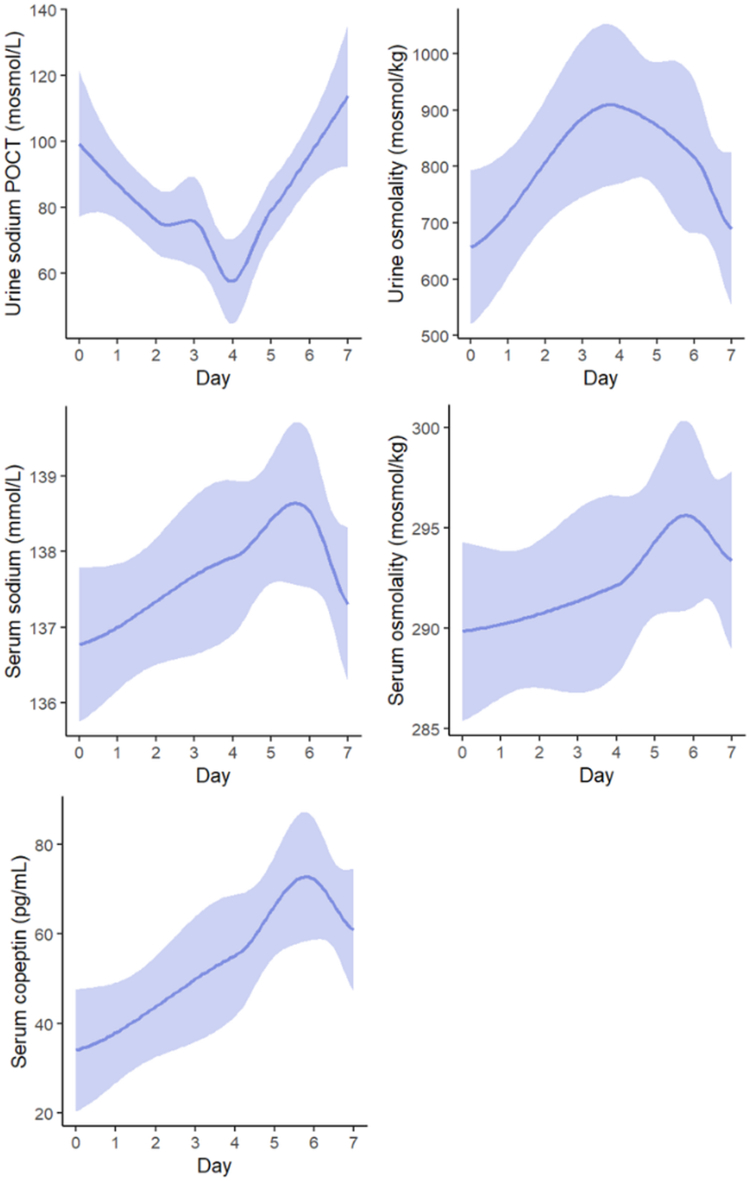
Development of serum copeptin and serum and urinary sodium concentration and osmolality over the course of the study. POCT, point-of-care testing.

Circulating blood volume and tonicity are primarily regulated by the sympathetic nervous system, the RAAS and AVP. Temporal changes of crucial parameters in this context are visualized in Figure 4 and summarized in Table 1. Compared to baseline values, an increase in serum osmolality (+5.69 mosmol/kg ± 5.88, *P* = 0.004) and serum sodium (+1.77 mmol/l ± 2.46, *P* = 0.040) was observed and peaked on day 6. Serum potassium decreased to a mean nadir of 3.71 mmol/l ± 0.36 with a mean difference of −0.6 mmol/l ± 0.47 on day 4 as compared to baseline values (*P* < 0.001). Continuous urinary self-measurements via point-of-care testing displayed a transient U-shaped decrease of urinary sodium concentrations with a mean nadir on day 4 (Figure 4). Urinary osmolality peaked on day 4 with a mean increase of +250.08 mosmol/kg ± 302.11 (*P* = 0.044), whereas urinary sodium concentrations (point-of-care testing) reached a nadir on day 4 (57.63 mmol/l ± 43.35, *P* = 0.022 compared to baseline). The urinary sodium-to-potassium ratio followed this U-shaped course, reflecting renal sodium reabsorption and potassium excretion as opposed processes. Serum copeptin more than doubled during the ride from a mean baseline value of 34.03 pg/ml ± 18.46 to 72.31 pg/ml ± 28.76 on day 6 (*P* < 0.001). The observed increase of serum osmolality largely resulted from an increase in serum sodium.

No other obvious cause of peripheral edema could be detected. Kidney function, as assessed by the Chronic Kidney Disease Epidemiology Collaboration glomerular filtration rate equation (2021), cystatin C and 24-hour creatinine clearance, remained stable and within normal limits throughout the study. Additional biomarker testing for intrarenal injury using serum neutrophil gelatinase-associated lipocalin was unremarkable. No single case of acute kidney injury, defined as a serum creatinine increase ≥0.3 mg/dl, occurred. Mild hyponatremia was observed on 2 occasions (ID 3: 134 mmol/l, ID 9: 133 mmol/l) both on day 0. Colloid osmotic pressure, serum protein, and albumin decreased during the ride as a sign of hemodilution; however, overall colloid osmotic pressure remained within normal range in all participants during the study. Mean creatinine kinase values increased during the ride over the normal range and 1 participant (ID 3) showed a significant creatinine kinase increase greater than 8-fold the upper normal range (creatinine kinase max 1516 U/l, day 6) without clinical signs of rhabdomyolysis. Maximum creatinine kinase levels of all other participants remained less than 5-fold the upper normal range.

A multilevel model assessing the influence of total fluid intake (L per kg body weight) on several markers of the fluid volume status positively correlated with end-diastolic volume of the right ventricle (*beta* = 5.58, SD = 2.78, *t* = 2.10, *P* = 0.038) but not with changes of plasma volume (*P* = 0.086), NT-proBNP (*P* = 0.586), TBW per kg body weight (*P* = 0.135), ECW per kg body weight (*P* = 0.248) and end-systolic volume of the right atrium (*P* = 0.274). Further details on the multilevel model results are presented in Supplementary Table S7.

## Discussion

We report the results of a prospective observational study using a multimodal approach to investigate the effects of a 6-day bike ride on fluid and electrolyte balance and occurrence of peripheral edema in a cohort of 13 ultracyclists. Over the course of the bike ride, participants showed signs of extracellular fluid volume expansion as indicated by a significant increase in TBW, ECW, and plasma volume in the absence of relevant body weight losses. Although intravascular volume expansion induced cardiac volume overload as indicated by elevated NT-proBNP and nonsignificant increases in right atrial and ventricular volumes, extravasal volume increase was manifested as peripheral edema of the face and eyelids, and significant volume increases of ankles, wrists, index fingers, and thighs. Mechanistic studies to explain underlying processes revealed marked signs of sodium and water retention. Although urinary sodium retention peaked on day 4, effective osmolality and corresponding copeptin levels showed a steady rise until day 6.

Occurrence of peripheral edema in endurance sports other than ultracycling has been described in the literature and has not been investigated systematically.^7^^,^^8^^,^^20^ Fluid dynamics, including fluid volume expansion have been studied in endurance sports back in the 1980s and 1990s^21^^,^^22^; however, these observations may not be applicable for ultraendurance sports, especially ultracycling, when strenuous physical stress lasts over multiple days with insufficient recovery periods. As recently reported, the majority (54.2%) of athletes experienced self-reported swelling symptoms during or after an ultradistance bike ride, with a mean symptom onset after 3.14 (± 1.56) days.^6^ Available research investigating changes of body composition in ultracycling is not fully consistent but studies of relatively short cycling ultraraces (< 3 days) typically show no increase^23^^,^^24^ or even a decrease^15^ of body water and plasma volume. In contrast and similar to our observations, a continuous increase in ECW and serum sodium concentration was observed in a multistage mountain bike race over 7 consecutive days.^25^ Relevance of metabolic water release due to a sustained degradation of energy substrates is increasing with exercise duration. Consequently, body mass losses up to 5% appear necessary to avoid overhydration in ultraendurance activity.^26^ In our cohort, body weight remained approximately stable, which for itself indicates increased TBW. However, the role of fluid intake in the observed volume expansion in our cohort remained inconclusive. Using a multilevel model, fluid intake correlated with an increase of end-diastolic volume of the right ventricle; however, there was no association with other markers of fluid volume status.

Exercise-associated cardiac volume overload, as indicated by transient increases in NT-proBNP and volumes of the right atrium and right ventricle in our cohort, is a well-established phenomenon in endurance sports and has repeatedly been described in the literature.^5^^,^^27^^,^^28^ Although a transient increase in cardiac dimensions and biomarkers usually resolves after a short resting period, extreme endurance exercise may be associated with long-term adverse electrical and structural remodelling.^29^ Recently, a U- or J-shaped dose-response curve between physical activity volumes and cardiovascular health outcomes was proposed.^27^

According to existing evidence, circumferences of extremities are expected to decrease during prolonged exercise due to a decrease in muscle density as a result of glycogen loss and degradation of subcutaneous adipose tissue.^8^^,^^24^^,^^30^ Therefore, the observed increase of ankle, wrist, and phalanx Dig. 2 circumferences in our cohort likely reflect mild, subclinical peripheral edema, whereas the volume increase of the thighs is rather nonspecific and might also be explained by an increased volume of muscle mass.

### Proposed Mechanism

**Figure 5:**
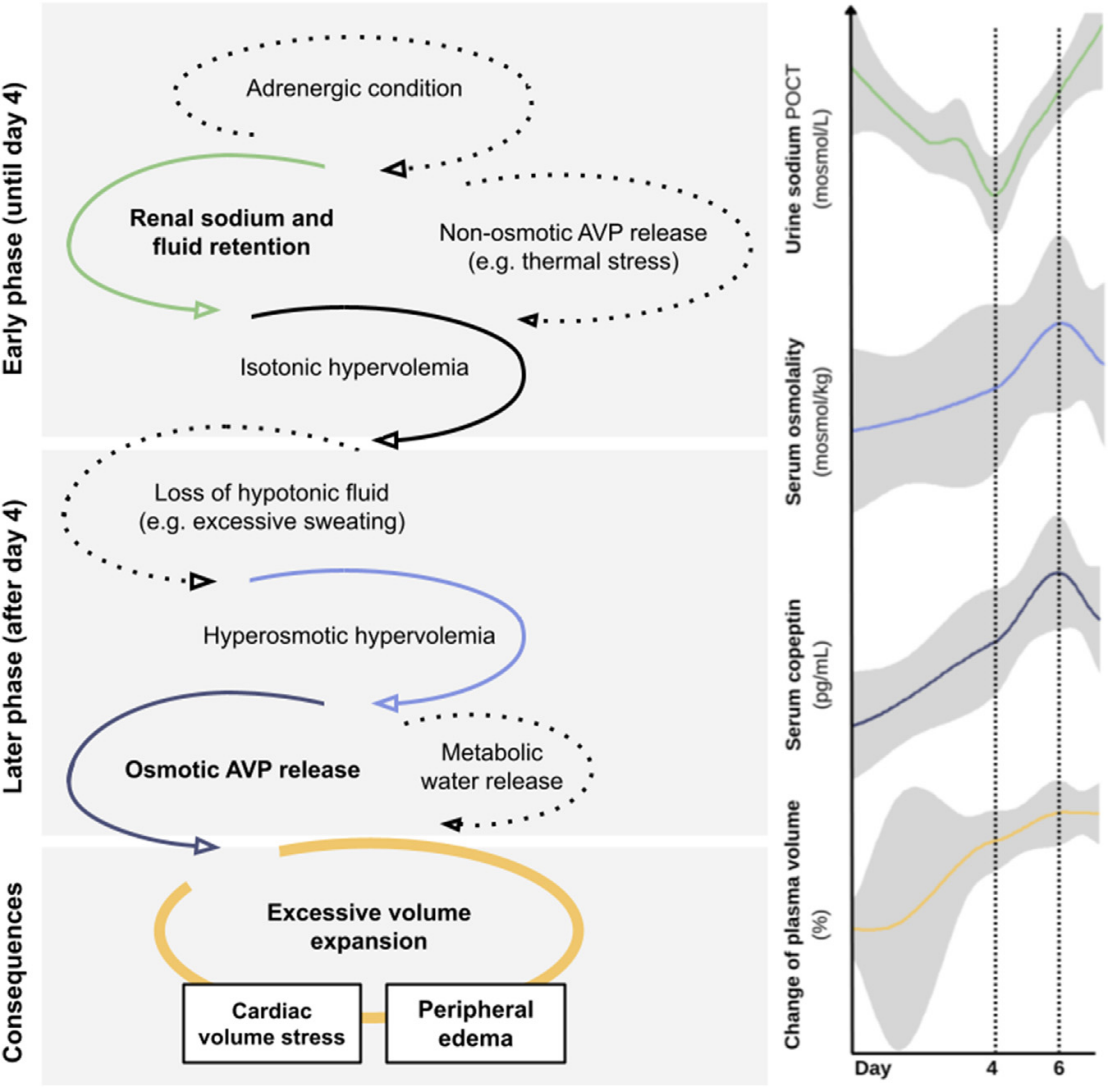
Proposed pathophysiological model of exercise-induced volume expansion in ultracycling. AVP, arginine vasopressin; POCT, point-of-care testing; RAAS, renin-angiotensin-aldosterone system. Solid arrows = as observed in our cohort, dotted arrows = as reported in literature.

Although the observational study design and the small cohort size do not allow for the conclusion of a definitive mechanistic explanation, several results of our study may help to better understand ongoing volume regulatory mechanisms in response to strenuous cycling over several days. A proposed pathophysiological model is illustrated in Figure 5.

Parasympathetic withdrawal and sympathetic activation are physiological initial responses of the autonomic nervous system to increase cardiac function and blood pressure at exercise onset. Consecutively, RAAS is activated, ensuring adequate tissue perfusion by aldosterone-mediated and angiotensin II-mediated sodium and water retention.^31^ The simultaneously decreasing urinary sodium concentration with increasing urinary osmolality observed in our cohort can be interpreted as indirect signs of renal aldosterone effects.^31^, ^32^, ^33^ This observation is underlined by a similarly U-shaped course of urinary sodium-to-potassium ratio, indicating aldosterone-mediated sodium reabsorption and potassium excretion.^34^ In addition, AVP release may be triggered initially by nonosmotic stimuli such as heat stress^35^ and/or arterial hypotension.^15^ Despite a continuously increasing TBW and plasma volume, we observed an increasing serum sodium concentration, possibly explained by a loss of hypotonic fluids (e.g., due to sweating). A rise in serum osmolality >290 mosmol/kg is mainly attributed to a rise in serum sodium that provides an adequate osmotic stimulus for AVP secretion (as measured by copeptin),^16^ further aggravating fluid volume. In fact, nonosmotic stimuli for AVP secretion (e.g., plasma volume contraction due to upright posture and muscle damage due to eccentric muscle contractions) may play a minor role in cyclists compared with runners.^15^

Considering that only 25% of gained ECW because of retention of sodium will remain in the intravascular space and the remaining 75% will distribute into the interstitial space, occurrence of peripheral edema is not surprising in conditions of significant extracellular fluid volume expansion. The mechanisms responsible for fluid accumulation appear to persist over time, and at least until day 6 (postride analysis). No steady state was reached during this period, as indicated by an almost linear increase in TBW, ECW, plasma volume, and NT-proBNP between day 0 to day 6. A minimum urinary sodium concentration on day 4 implies maximum RAAS activity and a declining RAAS activity thereafter. This might be explained by a beginning aldosterone escape, which is a physiological mechanism leading to increased sodium excretion in order to prevent further volume expansion.^36^ In contrast, a rise of serum copeptin and the corresponding effective osmolality was steadily increasing until postride analysis (day 6).

Previous research in marathon runners confirms that both aldosterone and AVP increase during exercise but return to baseline values at 22 hours after resting. Although plasma volume decreased directly after the run, an increase was shown at 22 hours.^22^ These findings confirm that fluid expansion occurs slowly, and protracted and sufficient recovery periods are crucial to avoid sustained increases of TBW.

### Limitations

This study was conceived as a pilot study to investigate basic principles of volume and electrolyte regulation, and assess the occurrence of peripheral edema in a small cohort of ultracyclists. The small cohort size clearly is the major limitation of the work, in the light of which no subgroup analyses, that is, investigating sex differences, could be performed and results of the multilevel regression analyses need to be interpreted carefully. A controlled study comparing different hydration protocols or strategies in a larger cohort would be required to clarify the impact of fluid intake on net hydration in ultracycling. RAAS activity was approximated by indirect markers (urinary sodium concentration, urinary sodium-to-potassium ratio) in our study. Separate analysis of serum renin and aldosterone were not performed because adequate preanalytical procedures (resting in a seated position for 30 minutes before blood collection) were not feasible in the study setting.^37^ Considering that signs of fluid retention were still increasing on postride analysis (day 6) and recovery was not completed (e.g., end-diastolic volume of the right ventricle), a longer study period and later recovery analysis would be an interesting setting for follow-up studies.

The photos were taken during the study visits by the study team or by the participants themselves during the ride, thus, a selection bias toward more “swollen faces” is possible. Another factor that needs to be considered is that the methods used for our study are based on equations that were validated in different populations. Respective results in our cohort may therefore only be interpreted with limitations. As an example, BIA is an indirect technique to determine body compartments by using regression equations that have been validated against a reference method in a specific population.^38^ Although BIA is widely used for body composition analysis in sports science, it lacks validity in athletes because of higher errors in conditions of significantly altered hydration.^38^ The use of tailored regression equations and further validation of BIA in athletes are therefore warranted.^39^

### Conclusion

Ultracycling over multiple days induces significant signs of fluid volume expansion (in particular an increase in TBW, ECW, and plasma volume) with cardiac volume overload (indicated by elevated NT-proBNP and a trend toward increasing right atrial and ventricular volumes) and peripheral edema. These changes are likely promoted by renal sodium and water retention. Recovery from these processes is not completed within 12 to 24 hours of resting. Athletes and health care professionals should be aware of this finding. Validation in a larger cohort (e.g., in a race setting) is needed.

## Disclosure

At the time of manuscript preparation, JSK and FRK were active ultradistance cyclists. PG reports grant support from Land Tirol during the conduct of the study and personal fees from UriSalt GmbH, outside the submitted work. AK received grants from CSL Vifor and Otsuka and consultancy fees from CSL Vifor, Otsuka, Catalyst Biosciences, Walden Biosciences, GlaxoSmithKline, and Delta4. JSK reports grant support from Land Tirol during the conduct of the study. SD received a congress support from Astra Zeneca. All the other authors have declared no conflicting interest.

## Supplementary Material

Materials and Methods.

Specific considerations on laboratory analyses.

Laboratory analyses (ZIMCL).

Point-of-care urinary testing (UriSalt).

Live tracking of body measurements, liquid intake, and photo documentation.

Bike ride statistics.

Bioelectrical impedance analysis.

Transthoracic echocardiography.

**Figure S1.** Timeline of the study course and procedures.

**Table S1.** Baseline and ride characteristics of study participants.

**Table S2.** Development of BIA parameters over the course of the study (excluding ID 11, *n* = 12).

**Table S3.** Change of plasma volume and serum NT-proBNP.

**Table S4.** Right atrial and ventricular volume changes as measured by transthoracic echocardiography.

**Table S5.** Swelling ratings of the face and eyes area on days 0, 4, 5, 6, and 7.

**Table S6.** Serially measured extremity circumferences.

**Table S7.** Multilevel model results with total fluid intake (l/kg body weight) as a predictor.

**Supplementary References**

**STROBE Statement** (PDF)
