# Supplementary material for: Exercise-Induced Fluid Retention, Cardiac Volume Overload, and Peripheral Edema in Ultra-Distance Cyclists
Source: Kidney Int Rep. 2023 Oct 31;9(1):152–61. doi: 10.1016/j.ekir.2023.10.025 (PMC10831365; doi:10.1016/j.ekir.2023.10.025)
Supplement: Supplementary File (PDF) [file mmc1.pdf]

Exercise-induced fluid retention, cardiac volume overload and peripheral edema in ultra-distance cyclists

Supplementary Information

Table of Contents

Materials and Methods..........2

Specific considerations on laboratory analyses..........2

Laboratory analyses (ZIMCL)..........2

Point-of-care urinary testing (UriSalt)..........3

Live tracking of body measurements, liquid intake and photo documentation..........3

Bike ride statistics..........4

Bioelectrical impedance analysis..........4

Transthoracic echocardiography..........4

Supplementary Tables..........5

Supplementary Table S1. Baseline and ride characteristics of study participants...........5

Supplementary Table S2. Development of *BIA parameters over the course of the study (excluding ID 11, n = 12).*..........1

Supplementary Table S3. Change of plasma volume and serum NT-proBNP...........1

Supplementary Table S4. Right atrial and ventricular volume changes as measured by transthoracic echocardiography...........2

Supplementary Table S5. Swelling ratings of the face and eyes area on days 0, 4, 5, 6 and 7. ... 2

Supplementary Table S6. Serially measured extremity circumferences...........2

Supplementary Table S7. Multilevel model results with total fluid intake (L / kg body weight) as a predictor..........3

Supplementary Figure..........4

Supplemental References..........4

STROBE Checklist..........5

Materials and Methods

The information on participants biological sex was obtained as self-reported.

Clinical visits were performed at baseline (day 0), interim (day 4), immediately post-ride (day 6) and after 12-24 hours of recovery (day 7). Laboratory (blood and urine) analyses and bioelectrical impedance analyses (BIA) were performed on the same days, including a 24-hour urine collection on days 1-2 and 6-7. Transthoracic echocardiography (TTE) was performed on days 0, 6 and 7. Throughout the entire ride, participants tracked fluid intake and self-measured body part circumferences in a custom mobile application (web-crossing GmbH). Continuous self-testing of urinary electrolytes was performed using a point-of-care testing (POCT) device (UriSalt GmbH).

Specific considerations on laboratory analyses

Copeptin is released in equimolar amounts from the same pre-pro-hormone as AVP but can be easily measured in plasma providing a reliable surrogate marker for AVP.[S1] Copeptin was analyzed by an enzyme-linked immunosorbent assay (ELISA) as described by the manufacturer (ABIN6955071, antibodies-online GmbH, PO Box 5201; Limerick, PA 19468, USA). All other laboratory analyses were performed at the ISO 15189 accredited Central Institute of Medical and Chemical Laboratory Diagnostics (ZIMCL). Changes of plasma volume were calculated indirectly by changes of hemoglobin concentrations and hematocrit, as previously described.[S2]

Laboratory analyses (ZIMCL)

Blood and urine samples were collected and promptly analyzed with fully automated tests in the *Central Institute for Medical and Chemical Laboratory Diagnosis* (ZIMCL) of the Innsbruck University Hospital, which undergoes regular internal and external quality controls and evaluations. Sodium and potassium were determined by indirect potentiometry; creatinine kinase and creatinine by enzymatic reactions; cystatin C, neutrophil gelatinase-associated lipocalin, and c-reactive protein by means of particle enhanced turbidimetric immunoassays; total protein via biuret reaction; albumin (by turbidimetry), and high sensitive TroponinT (by electrochemolumineszence immunoassay) on a Roche cobas 6000 system (neutrophil gelatinase-associated lipocalin and urine samples) or Roche

cobas 8000 system (all other parameters). Osmolality was determined by cryoscopy (FISKE 2400 osmometer, Fiske associates, Norwood, MA, USA) and colloid osmotic pressure by onkometry (Osmomat 050, Gonotec GmbH, Berlin, Germany)

On day 0 (until day 1) and on day 6 (until day 7), 24-hour urine collection was performed. Participants were instructed to collect all urine within the collection period into provided containers. Collection period started after voiding for spot urine collection (day 0 und day 6) and ended 24 hours later.

Point-of-care urinary testing (UriSalt)

Point-of-care self-testing of urinary sodium concentrations was conducted using a novel handheld device provided by *UriSalt GmbH*. Each participant was equipped with a testing device, sufficient test-stripes and a collection cup. After a training session on day 0, participants were capable of performing continuous urinary self-testing throughout the study period.

Live tracking of body measurements, liquid intake and photo documentation

A custom mobile application was designed in cooperation with *web-crossing GmbH* to facilitate live tracking of study relevant parameters during the bike ride. In order to ensure data protection and technical stability, each participant was equipped with a smartphone (*Huawei p30 light*) including the pre-installed Android-based application and received skill training to ensure reliable documentation.

During the pre-ride visit (day 0), measurement of body circumferences was first performed and explained by the study team using a standard measuring tape according to the following procedure: Circumferences of ankle (supramalleolar), mid-calf, thigh (10 cm proximal distance measured from patella), mid-upper arm, mid-forearm, wrist and mid-phalanx proximalis D2 of the right side of the body were measured. After the training, participants conducted self-measurements under supervision of the study team and re-training was performed in case of inaccuracy. During the bicycle ride, self-measurements were performed twice daily by the participants and documented in the mobile application.

Liquid intake was live-tracked via the mobile application using an intuitive user interface. Size (volume, mL) of individual drinking-bottles could be pre-defined and drunk volume was entered by a single touch selecting either one, half or quarter of the bottle.

Photo documentation of the face and the extremities was performed by the study team during the 4 clinical visits and during the ride by the participants themselves. Twenty physicians rated the photographs with respect to swelling of the eye region and the total face on a scale of 0 (“no swelling at all") to 5 (“heavy swelling”). The photos were all presented in random order, in grayscale, and in the same format.

Bike ride statistics

Each participant was equipped with a bike computer (*Wahoo ELMNT BOLT v1*) to track individual ride statistics, including covered distance, vertical meters and time on bike.

Bioelectrical impedance analysis

During the study visits, bioelectrical impedance analysis (BIA) was performed with each participant using *InBody 720 (InBody Europe B.V., Germany; software: LookinBody120.3.2.0.7)* in a standardized procedure. Measurement was performed in underwear and after bladder emptying. Due to a technical error that occurred in one patient (ID 11) on two visits, BIA results of this participant were excluded from further analysis.

Transthoracic echocardiography

Transthoracic echocardiography (TTE) was performed by an experienced cardiologist in the Echocardiography Department of the University Hospital Innsbruck according to current guidelines [Lang RM et al, Eur Heart J Cardiovasc Imaging, 2015 Mar; 16(3):233-70]. All images and echo-loops were acquired on a Phillips EPIQ CVx. The recorded echocardiograms were analysed with the IMAGE-COM® software from TOMTEC Imaging Systems

Supplementary Tables

Supplementary Table S1. Baseline and ride characteristics of study participants.

	Total, *Mean* (*SD*)	Participant (ID)												
		1	2	3	4	5	6	7	8	9	10	11	12	13
Sex		m	m	m	m	m	f	m	f	f	f	f	m	m
Age (year category)	33.38 (8.52)	20s	30s	30s	20s	30s	20s	50s	40s	20s	20s	30s	30s	30s
BSA (m²)	1.82 (0.16)	1.88	1.99	1.91	1.84	1.94	1.68	1.98	1.68	1.51	1.54	1.92	1.76	1.96
Overall distance (km)	1205.40 (220.10)	1150 .74	1258.96	1303.70	1383.08	817.93	1632.46	806.18	1253.35	1371.23	1072.46	1058.83	1208.22	1353.07
Overall climb (m)	19417 (4064)	2384 7	18565	23435	18508	17766	29242	15602	21278	17338	18232	12259	18133	18211
Hours on the bike	58.39 (7.93)	60.8 0	55.95	58.83	58.12	45.43	79.43	49.42	65.10	59.03	58.03	50.82	61.28	56.87
Training status[a], distance / climb (km)	14710 /200.7	1100 0 /93.0	12000 /180.0	14000 /220.0	15000 /100.0	25000 /500.0	22826 /284.4	6500 /40.0	15000 /150.0	17000 /300.0	10000 /204.0	12000 /40.0	15000 /150.0	16000 /240.0
Daily fluid intake (l)	4.69 (1.62)	4.31 (1.11)	5.34 (1.04)	6.15 (1.72)	6.80 (1.34)	3.68 (1.18)	4.95 (1.46)	3.94 (1.00)	2.71 (0.42)	4.69 (1.53)	3.90 (0.43)	4.37 (1.35)	4.31 (0.70)	6.06 (1.62)

m, male; f, female; BSA, body surface area; [a] during the previous calendar year (2020)

Supplementary Table S2. Development of *BIA parameters over the course of the study (excluding ID 11, n = 12).*

	min/max	day<br>0 (pre)	4 (interim)	6 (post)	7 (recovery)
Weight (kg)	*51.20/ 82.50*	68.18 (10.28)	68.08 (10.20)	68.00 (9.86)	68.10 (10.12)
SMM (kg)	*23.70/ 45.70*	33.92 (5.88)	35.03 (5.87)	35.29 (5.81)	34.89 (6.03)
Fat (kg)	*2.10/ 15.40*	8.43 (3.30)	6.10 (2.23)	5.39 (2.44)	6.48 (2.79)
TBW (L)	*31.30/ 57.60*	44.11 (7.18)	45.54 (7.30)	46.08 (7.16)	45.33 (7.25)
ECW (L)	*11.60/ 21.80*	16.71 (2.63)	17.22 (2.72)	17.51 (2.59)	17.21 (2.61)

SMM; skeletal muscle mass; TBW, total body water; ECW, extracellular water
Mean and standard deviation (parentheses) are displayed.

Supplementary Table S3. Change of plasma volume and serum NT-proBNP.

	min /max	day<br>0 (pre)	4 (interim)	6 (post)	7 (recovery)
Change of plasma volume (%)	*0.01 0.39*	ref	*14.11 (11.16)*	18.86 (10.71)	18.67 (8.69)
NT-proBNP (ng/L)	*25.00/ 820.00*	34.46 (18.87)	290.39 (248.79)	332.45 (217.06)	100.54 (76.33)

NT-proBNP, N-terminal pro brain natriuretic peptide;
Mean and standard deviation (parentheses) are displayed.

Supplementary Table S4. Right atrial and ventricular volume changes as measured by transthoracic echocardiography.

	min/ max	day 0 (pre)	4 (interim)	6 (post)	7 (recovery)
RAESV (mL)	*32.4/ 87.00*	54.21 (11.25)	*NA*	63.84 (13.18)	54.00 (14.31)
RVEDV (mL)	*44.82/ 102.49*	71.09 (13.98)	*NA*	79.71 (15.23)	80.11 (14.42)

RAESV, right atrial end-systolic volume; RVEDV, right ventricular end-diastolic volume
Mean and standard deviation (parentheses) are displayed.

Supplementary Table S5. Swelling ratings of the face and eyes area on days 0, 4, 5, 6 and 7.

	Mean	day 0 (pre)	4 (interim)	5	6 (post)	7 (recovery)
Face	2.31 (0.56)	2.29 (0.35)	2.64 (0.50)	3.06 (0.60)	2.07 (0.55)	2.38 (0.58)
Eyelids	2.40 (0.65)	2.08 (0.51)	2.94 (0.67)	2.94 (0.79)	2.26 (0.54)	2.46 (0.46)

Mean and standard deviation (parentheses) are displayed.

Supplementary Table S6. Serially measured extremity circumferences.

	Mean	day 0 (pre)	4 (interim)	6 (post)	7 (recovery)
Upper arm (cm)	*27.73 (2.58)*	27.45 (2.63)	27.97 (2.73)	27.62 (2.41)	27.86 (2.55)
Forearm (cm)	*25.03*	24.82	25.01	25.12	25.15

	(2.64)	(2.99)	(2.61)	(2.52)	(2.45)
Wrist (cm)	*16.73*	16.57	16.77	16.76	16.82
	(1.30)	(1.20)	(1.38)	(1.19)	(1.43)
Phalanx D2 (cm)	*6.84*	6.73	6.79	6.92	6.90
	(0.68)	(0.61)	(0.73)	(0.69)	(0.68)
Thigh (cm)	*46.69*	45.95	47.26	46.61	46.93
	(3.45)	(3.88)	(3.16)	(3.14)	(3.61)
Ankle (cm)	*22.64*	22.46	22.41	22.92	22.78
	(1.23)	(0.96)	(1.36)	(1.37)	(1.24)

Mean and standard deviation (parentheses) are displayed.

Supplementary Table S7. Multilevel model results with total fluid intake (L / kg body weight) as a predictor.

Independent variable	*b*	*SD*	*df*	*t*	*p*
Δ change of plasma volume (%)	4.08	2.35	90	1.74	.086
Δ serum NT-proBNP (ngl)	-16.22	29.69	90	-0.55	.586
Δ total body water / body weight	-0.00	0.00	83	-1.51	.135
Δ extracellular water / body weight	0.00	0.00	83	1.16	.248
Δ RVEDV	5.85	2.78	90	2.10	.038*
Δ RAESV	2.11	1.92	90	1.10	.274

NT-proBNP, N-terminal pro brain natriuretic peptide; RAESV, right atrial end-systolic volume; RVEDV, right ventricular end-diastolic volume.
**Level of significance: $p < 0.05$.*

Note. The investigated multilevel models considered individual starting points of each measure (random intercepts). We analyzed these models using the R package nlme.[18] All models fits were significant with intercepts p < .05.

Supplementary Figure

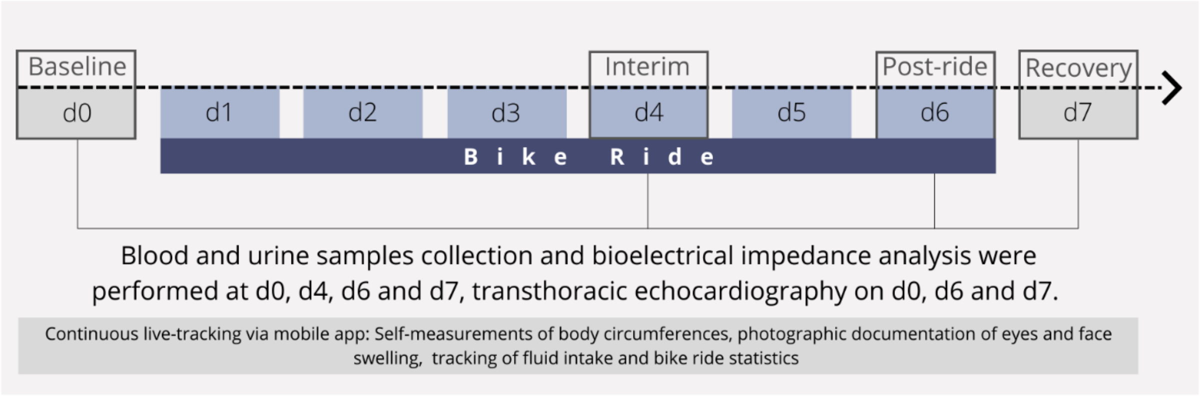

Supplementary Figure S1. Timeline of the study course and procedures.

d, day

Supplemental References

S1. Refardt J, Winzeler B, Christ-Crain M. Copeptin and its role in the diagnosis of diabetes insipidus and the syndrome of inappropriate antidiuresis. *Clin Endocrinol (Oxf)*. Jul 2019;91(1):22-32. doi:10.1111/cen.13991
S2. Dill DB, Costill DL. Calculation of percentage changes in volumes of blood, plasma, and red cells in dehydration. *J Appl Physiol*. Aug 1974;37(2):247-8. doi:10.1152/jappl.1974.37.2.247

STROBE Checklist

STROBE Statement—Checklist of items that should be included in reports of *cohort studies*

	Item No	Recommendation
**Title and abstract**	1 ✓	(*a*) Indicate the study's design with a commonly used term in the title or the abstract
	✓	(*b*) Provide in the abstract an informative and balanced summary of what was done and what was found
**Introduction**		
Background/rationale	2 ✓	Explain the scientific background and rationale for the investigation being reported
Objectives	3 ✓	State specific objectives, including any prespecified hypotheses
**Methods**		
Study design	4 ✓	Present key elements of study design early in the paper
Setting	5 ✓	Describe the setting, locations, and relevant dates, including periods of recruitment, exposure, follow-up, and data collection
Participants	6 ✓	(*a*) Give the eligibility criteria, and the sources and methods of selection of participants. Describe methods of follow-up
		(*b*) For matched studies, give matching criteria and number of exposed and unexposed
Variables	7 ✓	Clearly define all outcomes, exposures, predictors, potential confounders, and effect modifiers. Give diagnostic criteria, if applicable
Data sources/ measurement	8* ✓	For each variable of interest, give sources of data and details of methods of assessment (measurement). Describe comparability of assessment methods if there is more than one group
Bias	9 ✓	Describe any efforts to address potential sources of bias
Study size	10 ✓	Explain how the study size was arrived at
Quantitative variables	11 ✓	Explain how quantitative variables were handled in the analyses. If applicable, describe which groupings were chosen and why
Statistical methods	12 ✓	(*a*) Describe all statistical methods, including those used to control for confounding
	✓	(*b*) Describe any methods used to examine subgroups and interactions
	✓	(*c*) Explain how missing data were addressed
		(*d*) If applicable, explain how loss to follow-up was addressed
		(*e*) Describe any sensitivity analyses
**Results**		
Participants	13* ✓	(a) Report numbers of individuals at each stage of study—eg numbers potentially eligible, examined for eligibility, confirmed eligible, included in the study, completing follow-up, and analysed
		(b) Give reasons for non-participation at each stage
		(c) Consider use of a flow diagram
Descriptive data	14* ✓	(a) Give characteristics of study participants (eg demographic, clinical, social) and information on exposures and potential confounders
	✓	(b) Indicate number of participants with missing data for each variable of interest
	✓	(c) Summarise follow-up time (eg, average and total amount)
Outcome data ✓	15*	Report numbers of outcome events or summary measures over time
Main results ✓	16	(*a*) Give unadjusted estimates and, if applicable, confounder-adjusted estimates and their precision (eg, 95% confidence interval). Make clear which confounders were adjusted for and why they were included
		(*b*) Report category boundaries when continuous variables were categorized
		(*c*) If relevant, consider translating estimates of relative risk into absolute risk for a meaningful time period

Other analyses	✓	17	Report other analyses done—eg analyses of subgroups and interactions, and sensitivity analyses
**Discussion**			
Key results	✓	18	Summarise key results with reference to study objectives
Limitations	✓	19	Discuss limitations of the study, taking into account sources of potential bias or imprecision. Discuss both direction and magnitude of any potential bias
Interpretation	✓	20	Give a cautious overall interpretation of results considering objectives, limitations, multiplicity of analyses, results from similar studies, and other relevant evidence
Generalisability		21	Discuss the generalisability (external validity) of the study results
**Other information**			
Funding	✓	22	Give the source of funding and the role of the funders for the present study and, if applicable, for the original study on which the present article is based

*Give information separately for exposed and unexposed groups.

Note: An Explanation and Elaboration article discusses each checklist item and gives methodological background and published examples of transparent reporting. The STROBE checklist is best used in conjunction with this article (freely available on the Web sites of PLoS Medicine at http://www.plosmedicine.org/, Annals of Internal Medicine at http://www.annals.org/, and Epidemiology at http://www.epidem.com/). Information on the STROBE Initiative is available at http://www.strobe-statement.org.
